# Cost-effectiveness of child caries management: a randomised controlled trial (FiCTION trial)

**DOI:** 10.1186/s12903-020-1020-1

**Published:** 2020-02-10

**Authors:** Tara Homer, Anne Maguire, Gail V. A. Douglas, Nicola P. Innes, Jan E. Clarkson, Nina Wilson, Vicky Ryan, Elaine McColl, Mark Robertson, Luke Vale

**Affiliations:** 1 Population Health Sciences Institute, Newcastle University, Newcastle upon Tyne, NE2 4AX UK; 2 School of Dental Sciences, Faculty of Medical Sciences, Newcastle University, Newcastle upon Tyne, UK; 3 Dental School, University of Leeds, Leeds, UK; 4 School of Dentistry, University of Dundee, Dundee, UK; 5 Dental Health Services Research Unit, University of Dundee, Dundee, UK

**Keywords:** Economic evaluation, Caries, Caries treatment, Clinical studies/trials, Pediatric dentistry, Dental public health

## Abstract

**Background:**

A three-arm parallel group, randomised controlled trial set in general dental practices in England, Scotland, and Wales was undertaken to evaluate three strategies to manage dental caries in primary teeth. Children, with at least one primary molar with caries into dentine, were randomised to receive Conventional with best practice prevention (C + P), Biological with best practice prevention (B + P), or best practice Prevention Alone (PA).

**Methods:**

Data on costs were collected via case report forms completed by clinical staff at every visit. The co-primary outcomes were incidence of, and number of episodes of, dental pain and/or infection avoided. The three strategies were ranked in order of mean cost and a more costly strategy was compared with a less costly strategy in terms of incremental cost-effectiveness. Costs and outcomes were discounted at 3.5%.

**Results:**

A total of 1144 children were randomised with data on 1058 children (C + P *n* = 352, B + P *n* = 352, PA *n* = 354) used in the analysis. On average, it costs £230 to manage dental caries in primary teeth over a period of up to 36 months. Managing children in PA was, on average, £19 (97.5% CI: -£18 to £55) less costly than managing those in B + P. In terms of effectiveness, on average, there were fewer incidences of, (− 0.06; 97.5% CI: − 0.14 to 0.02) and fewer episodes of dental pain and/or infection (− 0.14; 97.5% CI: − 0.29 to 0.71) in B + P compared to PA. C + P was unlikely to be considered cost-effective, as it was more costly and less effective than B + P.

**Conclusions:**

The mean cost of a child avoiding any dental pain and/or infection (incidence) was £330 and the mean cost per episode of dental pain and/or infection avoided was £130. At these thresholds B + P has the highest probability of being considered cost-effective. Over the willingness to pay thresholds considered, the probability of B + P being considered cost-effective never exceeded 75%.

**Trial registration:**

The trial was prospectively registered with the ISRCTN (reference number ISRCTN77044005) on the 26th January 2009 and East of Scotland Research Ethics Committee provided ethical approved (REC reference: 12/ES/0047).

## Background

Dental caries has a large health and economic impact for the United Kingdom (UK) as it is the most common disease in children [1–4]. Treating oral disease is expensive, costing NHS England £3.4 billion annually [5].

In the UK there is uncertainty surrounding the best strategy to manage caries in primary teeth, especially in primary care. There is debate about the clinical- and cost-effectiveness of conventional restorations (removing a carious lesion with a drill and placement of a restoration) compared to minimally-invasive biologically-orientated strategies (sealing-in a carious lesion with an adhesive restoration or preformed metal crown rather than removing it), or prevention-focused strategies [6–9].

Cost-effectiveness analysis allows treatment comparisons in terms of both costs and effects [10]. Recent cost-effectiveness analyses of managing dental caries found the Hall Technique (HT), a method for managing carious lesions by sealing-in, to be cost-effective compared to conventional restorations [11] and compared to both conventional restorations and a Non-Restorative Cavity Control approach [12]. However, these studies followed outcomes on single teeth and have focused on one type of biological approach (i.e. HT).

A large trial, FiCTION (Filling Children’s Teeth: Indicated or Not?), was undertaken to measure the costs and effects, in terms of dental pain and/or infection, of three strategies to manage dental caries in the primary teeth of young children with dentine caries in the UK [13]. The strategies evaluated were Conventional restorations with best practice prevention (C + P), Biological management of carious lesions with best practice prevention (B + P), and best practice Prevention Alone (PA). The C + P strategy involved the complete mechanical removal of carious tooth tissue using local anesthesia and a drill followed by placement of a restoration alongside best practice preventive therapy and has been considered standard practice in the management of dental caries [14, 15]. The B + P strategy involved sealing-in carious lesions using a variety of techniques including adhesive restorative materials or preformed metal crowns placed using the HT along with preventive therapy; Schwendicke et al.’s (2018, 2019) analyses focused on the HT component of B + P [11, 12]. PA involved avoiding restorative intervention and using four components of preventive management; toothbrushing (with toothpaste of at least 1000ppmF concentration), dietary advice, fluoride varnish application, and fissure sealants to prevent further carious lesions.

The trial methodology and clinical outcomes are presented elsewhere [13, 16, 17]. In brief, this multi-center, three-arm, parallel group, patient-randomised controlled trial set in general dental practices in England, Scotland, and Wales was undertaken to evaluate three strategies to manage caries in the primary teeth of children aged 3 to 7 years with at least one primary molar tooth with caries lesions extending into dentine. The original planned follow-up was 3 years but due to an extension in the recruitment period this was revised to an average target follow-up of 35.5 months (a minimum of 23 months and a maximum of 36 months).

## Methods

Reporting for this study follows the Consolidated Health Economic Evaluation Reporting Standards (CHEERS) [18]. The trial was registered with the ISRCTN (reference number ISRCTN77044005) and East of Scotland Research Ethics Committee provided ethical approved (REC reference: 12/ES/0047).

### Data analyses

The economic evaluation was undertaken from the perspective of the healthcare provider in the UK, the National Health Service (NHS).

#### Estimation of costs

Time/materials-based costing was used to estimate the costs at every visit to manage dental caries in primary teeth. These costs depended on the quantity of dental care resources used for each child during their time in the trial (up to 36 months post-randomisation). Resource use data, to inform the cost analysis, were collected via case report forms (CRFs) completed by the clinician at every visit. Costs were categorised as staffing, preventive treatments, operative treatments (restoration materials), other associated items (e.g. radiographs), referrals, and prescriptions. Capital costs were excluded as all three strategies were provided as part of current care; therefore, these costs would have been incurred regardless of which strategy was implemented. Unit costs, based on the materials required for each treatment, were multiplied by the number of resources used. Unit costs are detailed in Additional file 1 and briefly described below. All costs are in 2018 pounds Sterling.

The length of time for each visit, based on the start and end time recorded in the CRF, was used to estimate dental personnel costs. Time spent providing prevention was subtracted from total visit time to take into account that the same personnel may not provide preventive and operative treatments. We assumed a dental nurse would be present for the full duration of each visit.

Preventive care was integral to all three arms and was expected to be provided regardless of randomised allocation. Preventive treatment costs were the resources used for fluoride applications and fissure sealants placed on first permanent molars.

Operative treatments were included in two arms; C + P (e.g. local anesthetic, carious tissue removal, and restoration) and B + P (e.g. partial/no carious tissue removal and restoration), but some treatments were included in all three arms (e.g. extractions under local anesthetic and pulp therapy). Information on the number of surfaces treated was also collected since treatment of more than one tooth surface could incur additional costs (e.g. additional restorative material). The cost of resources used at every visit were also included, regardless of treatment. Other treatment costs included radiographs and inhalation sedation.

A patient referral was reported if a child was referred to a dental hospital/clinic for a consultation and/or operative treatment. The costs associated with referrals were categorised A-F (see Additional file 2) depending on treatment provided, where it took place, who provided it, and the number of visits required.

#### Estimation of effects

The original primary outcome, incidence of dental pain and/or infection was modified during the trial to include a co-primary outcome, number of episodes of dental pain and/or infection. Number of episodes was included as it was considered more clinically relevant and statistically more sensitive to analyse the frequency of dental pain and/or infection experienced by a child.

Incidence is defined as the proportion of children with at least one episode of dental pain and/or infection during their time in the study. Episodes were defined on a tooth-by-tooth basis based on the frequency of dental pain and/or infection reported during the child’s follow-up. However, if multiple teeth had dental pain and/or infection at the same visit, this was counted as one episode or if the same tooth had dental pain and/or infection at consecutive visits, this was counted as one episode regardless of the time between visits [13]*.* Data on dental pain due to dental caries and clinically diagnosed infection were collected on the CRF at every visit. It was assumed that those who did not have regular appointments did not need further treatment and/or did not experience dental pain and/or infection.

#### Cost-effectiveness analysis

The economic analysis was conducted on the basis of intention-to-treat (ITT). Children were included in the ITT analysis if they had at least one CRF and therefore at least one clinical assessment of the primary outcome. The economic analysis compared the three strategies in terms of mean costs and effects over the follow-up period. Both costs and effects were discounted at the recommended rate of 3.5% [19]. Effects were discounted, based on when the incidence or episode of dental pain and/or infection began. To enable the estimation of budget impact [20] the average total costs by cost category presented in Table 1 were not discounted.

**Table 1:** Average total cost (£) per child by strategy ^a^

	Total cost per child (£)		
	C + P	B + P	PA
Resource	Mean [SD]	Mean [SD]	Mean [SD]
Staff costs	18.78 [6.07]	18.28 [6.27]	17.36 [5.95]
Prevention costs	0.66 [0.76]	0.78 [0.88]	0.81 [0.88]
Operative treatment costs	8.18 [6.72]	7.84 [5.96]	4.09 [4.05]
Other treatments costs	0.66 [2.56]	0.47 [1.84]	0.52 [1.90]
Referral costs	5.22 [23.35]	4.96 [23.65]	10.23 [43.81]
Prescription costs	0.07 [0.29]	0.04 [0.14]	0.08 [0.32]
Total practice level treatment cost (exc. referrals) per child per visit	28.36 [11.08]	27.40 [10.81]	22.86 [8.11]
Total treatment cost per child	250.48 (221.70)	231.27 (214.47)	211.32 (257.28)

^a^costs are not discounted in this table but presented in the common price year to allow for budget impact

For the incremental analysis, the strategies were ranked in terms of increasing mean cost and a more costly strategy was compared with a less costly strategy in terms of incremental cost-effectiveness. A treatment was considered to be dominated if it was more costly and less effective than its comparator [10]. If a treatment was not dominated, an incremental cost-effectiveness ratio (ICER) was estimated. The ICER is the difference in mean costs divided by the difference in mean effects and gives an estimate of the mean cost per additional unit of effect [10].

STATA software was used for all analyses [21]. Regressions on costs and effects were run simultaneously using seemingly unrelated regression (SUR) [22]. SUR permits the simultaneous estimation of costs and effects, calculated at an individual level, while accounting for unobserved individual characteristics that could affect both costs and effects and lead to potential correlation between these two dependent variables [23]. In addition, the SUR controlled for additional covariates (age, time in study, and practice variation) that may affect costs, effects, or both.

A stochastic sensitivity analysis, using the bootstrapping technique [24], explored the impact of the statistical imprecision surrounding estimates of costs, effects, and cost-effectiveness. The bootstrapped results from the incremental analysis were used to estimate net benefits (NB). The NB statistic is given by:

$$ \mathrm{NB}=\left(\uplambda \times \Delta \mathrm{e}\right)\hbox{--} \Delta \mathrm{c} $$

where λ is the willingness to pay threshold, Δ is the difference between a strategy and its comparator (i.e. least costly strategy), e are the mean effects, and c are the mean costs [10]. A strategy is considered to be cost-effective if NB > 0 or, when more than two strategies are compared, a strategy which has the highest NB at a given threshold value for society’s willingness to pay for a unit of oral health benefit. As there is no nationally or internationally agreed willingness to pay threshold to avoid dental pain and/or infection an arbitrary threshold of £1000, used by O’Neill et al. (2017), was adopted for this analysis [25]. A cost-effectiveness frontier [26] was generated to illustrate uncertainty by showing which strategy was likely to have the highest NB over a range of different willingness to pay values.

## Results

A total of 1144 children were randomised and data on 1058 children were used in the economic analysis (*n* = 86 children did not have any clinical assessment of the primary outcome and were not included in the ITT analysis). The children included in the economic analysis were evenly distributed across the three arms in terms of numbers randomised and baseline characteristics; 352 randomised to B + P, 352 to C + P, and 354 to PA. On average, children were 6 years old [sd: 1.3] when recruited and there was an even split between females (51%) and males (49%). The CONSORT (Consolidated Standards of Reporting Trial) flow diagram is provided in Additional file 6 but additional baseline characteristics, and clinical findings are presented elsewhere [13]. The median follow-up was 33.8 months (IQR 23.8, 36.7).

The percentage of missing data for the economic analysis was low (< 5%). There were 7713 visits recorded across the three arms. On average, children had seven visits during their time in the trial, each lasting 21 min. All three strategies were similar in terms of average number of visits (mean visits [sd]: C + P 7.7 [4.2], B + P 7.4 [4.1], and PA 6.8 [3.7]) and duration of visits (mean minutes [sd]: C + P 21.8 [6.9], B + P 21.2 [7.2], and PA 20.1 [6.7]).

The number of visits at which preventive treatment was provided was similar across the three arms with slightly more prevention provided in the PA arm (C + P 79%, B + P 79%, and PA 85% of visits). The three strategies differed in the frequency of operative treatments provided, with less than 20% of all PA visits involving operative treatment compared to over 40% of B + P and C + P visits. The type of operative treatment provided also differed, as would be expected given the nature of the different strategies.

A total of 96 children (C + P *n* = 31, B + P *n* = 31, and PA *n* = 34 children) were referred on 107 occasions for additional consultations and/or further treatment (C + P *n* = 32, B + P *n* = 36, and PA *n* = 39 referrals) resulting in 52 general anesthetics (GA) being undertaken (C + P *n* = 15, B + P *n* = 12, PA *n* = 25 referrals with GA). Four children did not attend their referral appointment (*n* = 1 C + P, *n* = 3 PA).

Table 1 summarizes the average cost per child per visit for the three strategies.

On average, it cost £230 to manage dental caries in a young child with at least one primary tooth with a dentinal carious lesion over a period of up to 36 months. On average, C + P was the most costly and PA was the least costly strategy. Staff time, operative treatments, and patient referrals were the main cost drivers. As expected, C + P and B + P incurred more operative treatment costs compared to PA.

As PA was, on average, the least costly strategy we compared this to B + P, the next costly strategy, and lastly C + P was included in the comparison. In terms of effectiveness, there was no evidence of a difference in incidence, or in episodes, of dental pain and/or infection between the three strategies. Table 2 summaries the results of the incremental analysis.

**Table 2:** Cost-effectiveness analysis for the comparison of PA vs B + P vs C + P ^a^

Investigation strategy	Cost [£] [97.5% CI]^b^	Incremental cost [£] [97.5% CI]^b c^	Incidence [97.5% CI]^b^	Incremental incidence [97.5% CI]^b c^	ICER^c^ [£]
Incremental cost per incidence of dental pain and/or infection avoided					
PA (*n* = 354)	206 [176 to 237]		0.44 [0.39 to 0.50]		
B + P (*n* = 352)	226 [201 to 252]	19 [−18 to 55]	0.39 [0.33 to 0.45]	−0.058 [−0.14 to 0.02]	328
C + P (*n* = 352)	245 [219 to 271]		0.41 [0.35 to 0.47]		Dominated by B + P
Incremental cost per episode of dental pain and/or infection avoided					
Investigation strategy	Cost [£] [97.5% CI]^b^	Incremental cost [£] [97.5% CI]^b c^	Episodes [97.5% CI]^b^	Incremental episodes [97.5% CI]^b c^	ICER ^c^ [£]
PA (*n* = 354)	206 [176 to 237]		0.70 [0.58 to 0.82]		
B + P (*n* = 352)	226 [201 to 252]	19 [−18 to 55]	0.56 [0.46 to 0.67]	−0.143 [−0.29 to 0.01]	133
C + P (*n* = 352)	245 [219 to 271]		0.60 [0.49 to 0.71]		Dominated by B + P

^a^ costs and effects are discounted at 3.5%; ^b^ 97.5% CI was used as it adjusts for multiple comparisons and should be interpreted as if it were a 95% CI; ^c^ estimated based on adjusted analysis (*n* = 1057; *n* = 1 child missing information on age); ^d^ ICER = incremental cost-effectiveness ratio

B + P was, on average, more costly but more effective, in terms of both incidence of, and episodes of, dental pain and/or infection avoided, compared to PA. At a willingness to pay threshold of £330 we would consider B + P cost-effective to avoid an incidence and £130 to avoid an episode of dental pain and/or infection compared to PA. As C + P was, on average, more costly and less effective than B + P, in terms of both incidence of, and episodes of, dental pain and/or infection, it was dominated by B + P.

**Fig. 1:**
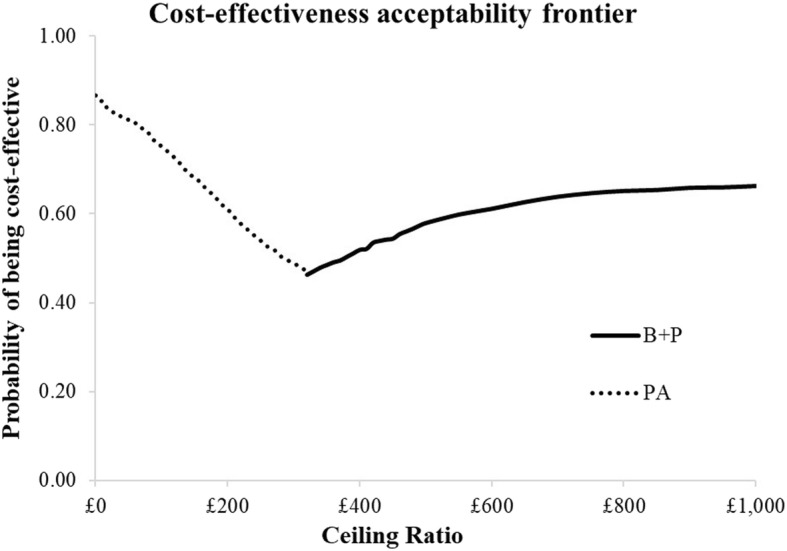
Probability of being cost-effective to avoid an incidence of dental pain and/or infection

Figure 1 illustrates uncertainty surrounding the point estimates in Table 2. The figure presents the strategy with the highest probability of being considered cost-effective at each willingness to pay threshold to avoid an incidence of dental pain and/or infection.

Figure 1 illustrates that PA would have the highest probability (87%) of being considered cost-effective if a decision were to be based on cost alone. However, as the willingness to pay threshold increases, the probability of B + P being considered cost-effective increases, but it never exceeds 65%. C + P would not be considered cost-effective compared to PA and B + P in this analysis.

**Fig. 2:**
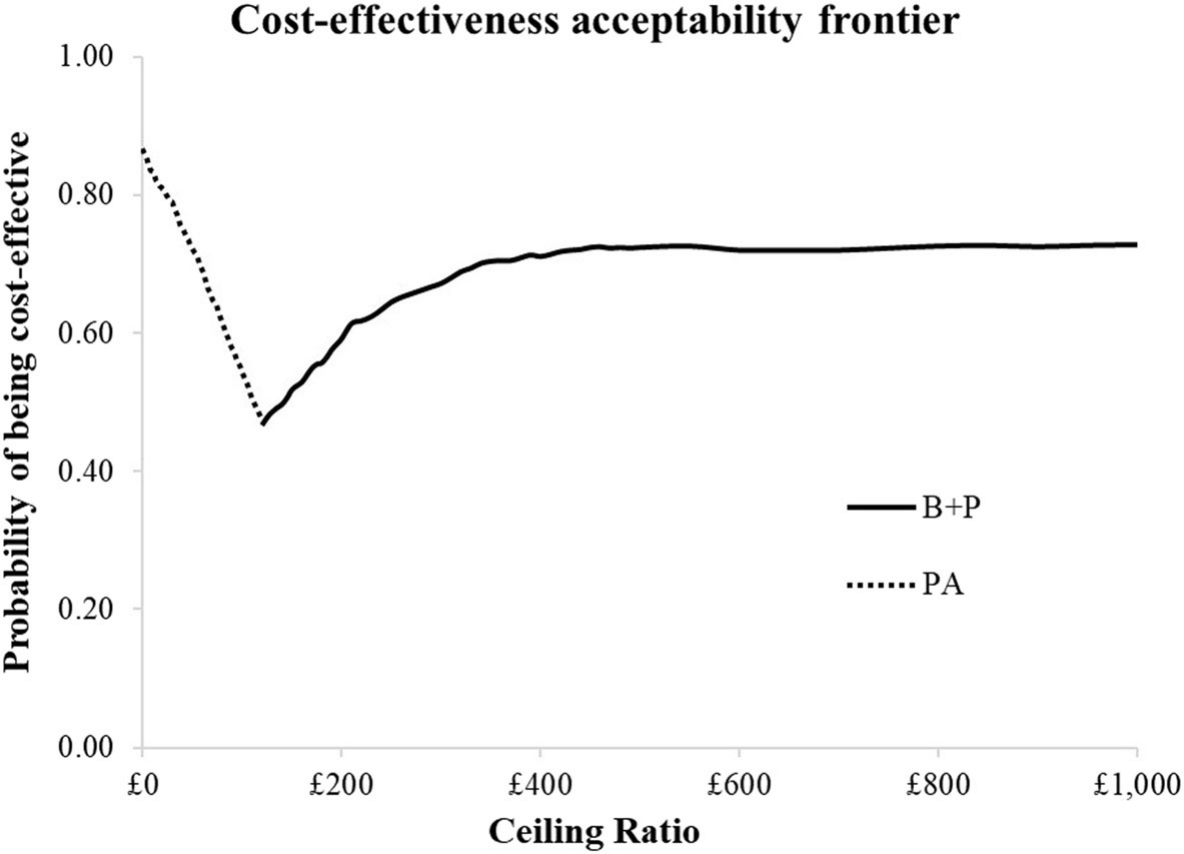
Probability of being cost-effective to avoid an episode of dental pain and/or infection

Figure 2 illustrates the strategy with the highest probability of being considered cost-effective at the different willingness to pay thresholds to avoid an episode of dental pain and/or infection.

In terms of episodes of dental pain and/or infection, the conclusions are similar except that B + P would be considered cost-effective at a lower willingness to pay threshold. The probability of B + P being considered cost-effective never exceeds 75%.

## Discussion

On average, it costs £230 to manage dental caries in primary teeth in a child with at least one tooth with a dentinal caries lesion over a period of up to 36 months. The main cost drivers were staff time, operative treatments, and patient referrals. On average, PA incurred a higher referral cost because that arm had more referrals and more referrals requiring GA, an important consideration in view of the morbidity associated with GA use [27–29].

Although in terms of cost-effectiveness PA was, on average, the least costly treatment, it was also the least effective for both incidence of, and episodes of, dental pain and/or infection. There was an 87% probability that PA would be considered cost-effective as the least costly option but B + P and C + P would, on average, provide more oral health benefits, albeit at a higher cost. If society was willing to pay £330 to avoid one additional child experiencing dental pain and/or infection, B + P would have the highest probability (47%) of being considered cost-effective compared to PA (46%) and C + P (7%). Similarly, when society is willing to pay £130 or more to avoid an episode of dental pain and/or infection, B + P would have the highest probability (49%) of being considered cost-effective compared to PA (45%) and C + P (6%).

Vermaire et al. (2014) and Samnaliev et al. (2015) came to similar conclusions in their analyses, in that treatments aimed at caries prevention increased the cost of providing treatment and that the opportunity cost of these treatments is dependent on the payers’ willingness-to-pay [30, 31]. Our results differ from other studies in which the HT, which was a component of our B + P intervention, was reported to be more effective and less costly [11, 12] when compared to conventional and preventive based strategies. However, both of these studies by Schwendicke et al. (2018, 2019) were based on treating a single tooth, or two contralateral teeth per child whereas in our study the whole child/mouth (up to 20 primary teeth per child) could be treated, a situation more representative of real life treatment provision [11, 12]. Our study also had considerably more data available to inform our analysis (*n* = 1058 children, *n* = 2721 teeth; compared with *n* = 142 children and teeth in Schwendicke et al. 2018; and *n* = 91 children, *n* = 182 teeth in Schwendicke et al. 2019) [11, 12]. The costs estimated in the two Schwendicke et al. (2018, 2019) studies were based on current charges to the health system [11, 12]. In the present analysis we based our costs on a very detailed costing exercise, however, when we used current charges to the NHS in a sensitivity analysis we still reached the same conclusion (see Additional files 3, 4, and 5). Schwendicke et al. (2019) found a negligible difference in total treatment costs between HT and conventional treatment and this difference only became clinically and statistically significant when patient costs were considered [11]. Parental time and travel costs to attend appointments were not considered in our analysis. Inclusion of such costs are unlikely to change our conclusions as the average number of visits and length of visits were similar across the arms. Costs incurred by the parent and child due to toothache, such as time off work, childcare, and time off school, were considered in a sensitivity analysis but did not change our overall conclusions. In terms of oral health effects, direct comparisons could not be made with previous studies [11, 12] which considered the pain associated with dental caries together with endodontic treatment and extractions. The main differences in our study are, firstly, that B + P encompassed a number of minimally-invasive restorations of which the HT was only one and secondly, treatment was at the participant level (including all primary teeth) and not at the single tooth level.

This economic analysis had a number of strengths and limitations. The main strength was that the analysis was pre-planned and the data used were collected as part of the trial. There were few missing data and all available data were included in the analysis despite the varying follow-up. A limitation of the analysis was that SUR model may not have been an appropriate fit for the co-primary outcomes. However, a trade-off was made between fitting the most appropriate model and applying one that allows for the correlation of costs and outcomes, which the SUR approach does. Finally, capital costs were excluded from the analysis; this omission reduced the total cost of each arm equally hence the incremental costs, ICER, and our overall conclusions remain unchanged.

In practical terms, we do not know society’s willingness to pay threshold to avoid dental pain and/or infection in a primary tooth. A judgement is required as to what value the NHS places on avoiding dental pain and/or infection. Recent research conducted by Lord et al. (2015) estimated the willingness to pay to avoid dental caries with pain in a primary tooth [32]. They estimated this to be £153 (95% CI: £93 to £213 – inflated to 2017) [33]. If we adopted this as the willingness to pay threshold the PA arm would have a 68% probability of being considered cost-effective compared to B + P (29%) and C + P (3%) in terms of an incidence of dental pain and/or infection avoided. A willingness to pay threshold to avoid an episode of dental pain and/or infection also needs to be determined but based on the Lord et al. (2015) threshold, B + P would have the highest probability (53%) of being considered cost-effective compared to PA (40%) and C + P (7%). Further research is needed to identify the most appropriate threshold to assess our results.

## Conclusions

To conclude, on average, PA is the least costly, despite having more referrals requiring GA, but the least effective strategy for managing dental caries in primary teeth. B + P has the potential to provide more oral health benefits to children with dentinal carious lesion in at least one primary molar tooth, however this comes at an additional cost. Over the willingness to pay values considered, the probability of B + P being considered cost-effective was approximately no higher than 65% to avoid an incidence of dental pain and/or infection and no higher than 75% to avoid an episode of dental pain and/or infection. It is unlikely that C + P would be considered cost-effective.

## Supplementary information

**Additional file 1.** “Unit costs” is a table summarising the unit costs used in the analysis.

**Additional file 2.** “Groupings for referrals” is a table summarising the different groupings used to categorise patient referrals.

**Additional file 3 **“Cost-effectiveness analysis for the comparison of PA vs B+P vs C+P arms based on fee-for-service costs in Scotland only (*n*=287)” is a the results of a sensitivity analysis which estimates costs based on charges to the NHS, based on the Scottish reimbursement rates (fee-for-service).

**Additional file 4. **“Cost-effectiveness analysis for the comparison of PA vs B+P vs C+P arms based on units of dental activity in England and Wales only costs (*n*=771)” is a the results of a sensitivity analysis which estimates costs based on charges to the NHS, based on the English reimbursement rates (Units of Dental Activity).

**Additional file 5. **“Cost-effectiveness analysis for the comparison of PA vs B+P vs C+P based on units of dental activity (England and Wales) and fee-for service (Scotland) costs (*n*=1058)” is a the results of a sensitivity analysis which estimates costs based on charges to the NHS, based on the both the Scottish and English reimbursement rates.

**Additional file 6.** “CONSORT flow diagram of participant journey through trial” illustrates the number of children screened, randomised, and included in the final analysis. This image is taken directly from Maguire et al. 2019.

## Data Availability

The datasets generated and analysed during the current study are not publicly available due to Regional Ethical Review Board regulations. Any reasonable requests for data should be sent to the corresponding author who, alongside the Trial Management Group, will review the request and give permissions.

## Abbreviations

B + P: Biological with best practice prevention
C + P: Conventional with best practice prevention
CHEERS: Consolidated Health Economic Evaluation Reporting Standards
CONSORT: Consolidated Standards of Reporting Trial
CRF: Case Report Form
FiCTION: Filling Children’s Teeth: Indicated or Not?
GA: General Anesthetics
HT: Hall Technique
HTA: Health Technology Assessment
ICER: Incremental cost-effectiveness ratio
IQR: Interquartile Range
ITT: Intention-to-treat
NB: Net Benefit
NHS: National Health Service
NIHR: National Institute of Health Research
PA: Best practice Prevention Alone
SD: Standard Deviation
SUR: Seemingly unrelated regression
UK: United Kingdom
