# Supplementary material for: Cost-effectiveness of child caries management: a randomised controlled trial (FiCTION trial)
Source: BMC Oral Health. 2020 Feb 10;20:45. doi: 10.1186/s12903-020-1020-1 (PMC7011536; doi:10.1186/s12903-020-1020-1)
Supplement: Supplementary file 1 — Additional file 1. “Unit costs” is a table summarising the unit costs used in the analysis. [file 12903_2020_1020_MOESM1_ESM.docx]

**Additional File 1**

**Table** Unit costs

| **Resource** | **Unit** | **Cost (£)** | **Source** |
| --- | --- | --- | --- |
| **Treatment Provider** | | | |
| GDP | Cost per min | £0.68 | CRF (Q10a/11a)/PSSRU 2017 |
| Dental Therapist | Cost per min | £0.28 | CRF (Q10a/11a)/Agenda for change Band 5 pt 20 |
| Dental Hygienist | Cost per min | £0.28 | CRF (Q10a)/Agenda for change Band 5 pt 20 |
| Oral health educator | Cost per min | £0.28 | CRF (Q10a)/Agenda for change Band 5 pt 20 |
| Childsmile/Extended duty dental nurse | Cost per min | £0.23 | CRF (Q10a)/Agenda for change Band 4 pt14 |
| Vocational therapist | Cost per min | £0.23 | CRF (Q10a)/Agenda for change Band 4 midpoint |
| Dental nurse | Cost per min | £0.21 | CRF (Q10a)/Agenda for change Band 4 pt 1 |
| CT1 | Cost per min | £0.35 | CRF (Q10a)/Agenda for change Band 6 pt 6/ Band 7 pt 1 |
| Dental nurse trainee | Cost per min | £0.16 | CRF (Q10a)/Agenda for change 75% of Band 4 pt 1 |
| **Resources used at every visit** | | | |
| Resources at every visit | Cost per visit | £2.19 | CRF completed |
| Resources at every visit – fluoride varnish | Cost per visit | £1.74 | CRF (Q10b) |
| **Prevention** | | | |
| Fissure Sealants of permanent teeth (resin) | Cost per visit | £3.99 | CRF (Q10b) |
| Fluoride Varnish | Cost per visit | £0.27 | CRF (Q10b) |

| **Resource** | **Unit** | **Cost (£)** | **Source** |
| --- | --- | --- | --- |
| **Operative Treatment** | | | |
| Topical anesthetic gel | Cost per visit | £0.05 | CRF (Q12) |
| Local anesthetic | Cost per visit | £1.08 | CRF (Q12) |
| Fissure sealant – glass ionomer | Cost per tooth | £4.54 | CRF (Q12) |
| Preformed metal crown | Cost per tooth | £14.48 | CRF (Q12) |
| Hall technique crown | Cost per tooth | £6.92 | CRF (Q12) |
| Pulpotomy | Cost per tooth | £25.02 | CRF (Q12) |
| Extraction | Cost per tooth | £2.58 | CRF Q(12) |
| Opening a lesion | Cost per tooth | £2.55 | CRF Q(12) |
| **Filling** | | | |
| Filling – amalgam | Cost per tooth | £4.11 | CRF (Q12) |
| Filling – glass ionomer | Cost per tooth | £4.54 | CRF (Q12) |
| Filling – composite | Cost per tooth | £3.48 | CRF (Q12) |
| Filling – compomer | Cost per tooth | £3.48 | CRF (Q12) |
| Filling – resin modified glass ionomer | Cost per tooth | £4.82 | CRF (Q12) |
| Filling – resources used to administer a filling | Cost per tooth | £5.10 | CRF (Q12) |
| **More than 1 surface used** | | | |
| Filling – amalgam | Cost per tooth | £4.27 | CRF (Q12) |
| Filling – glass ionomer | Cost per tooth | £5.09 | CRF (Q12) |
| Filling – composite | Cost per tooth | £4.04 | CRF (Q12) |
| **Resource** | **Unit** | **Cost (£)** | **Source** |
| Filling – compomer | Cost per tooth | £4.04 | CRF (Q12) |
| Filling – resin modified glass ionomer | Cost per tooth | £5.38 | CRF (Q12) |
| Filling – resources used to administer a filling | Cost per tooth | £5.38 | CRF (Q12) |
| **Miscellaneous** | | | |
| Radiographs | Cost per image | £1.51 | CRF (Q9) |
| Inhalation sedation (exc. staff costs) | Cost per visit | £59.76 | CRF (Q17)/NICE 2010 (inflated to current prices) |
| **Medications** | | | |
| Paracetamol | Cost per dose | £0.04 | CRF (Q20)/medicines complete |
| Ibuprofen | Cost per dose | £0.09 | CRF (Q20)/medicines complete |
| Mouth rinse | Cost per bottle | £7.14 | CRF (Q20)/medicines complete |
| Mouth spray | Cost per spray | £4.64 | CRF (Q20)/medicines complete |
| Mouth gel | Cost per tube | £1.56 | CRF (Q20)/medicines complete |
| Bonjela® | Cost per tube | £3.55 | CRF (Q20)/medicines complete |
| amoxicillin | Cost per dose | £0.05 | CRF (Q21)/medicines complete |
| penicillin | Cost per dose | £0.74 | CRF (Q21)/medicines complete |
| metronidazole | Cost per dose | £1.03 | CRF (Q21)/medicines complete |
| erythromycin | Cost per dose | £0.22 | CRF (Q21)/medicines complete |
| **Referrals** | | | |
| Grouping A | Cost per referral | £118.00 | Patient referral form/Personal communication |
| Grouping B | Cost per referral | £793.00 | Patient referral form//Personal communication |

| **Resource** | **Unit** | **Cost (£)** | **Source** |
| --- | --- | --- | --- |
| Grouping C | Cost per referral | £793.00 | Patient referral form//Personal communication |
| Grouping D | Cost per referral | £418.00 | Patient referral form//Personal communication |
| Grouping E | Cost per referral | £418.00 | Patient referral form//Personal communication |
| Grouping F | Cost per referral | £118.00 | Patient referral form//Personal communication |
| **Pain medication (over the counter)** | | | |
| Calprofen (ibuprofen)^a^ | Cost per bottle | 3.59 | Parent/Questionnaire/<http://www.lloydspharmacy.com/en/calprofen-174-ibuprofen-3-months-100ml> |
| Calpol (Paracetamol) ^a^ | Cost per bottle | 2.99 | Parent/Questionnaire/<http://www.lloydspharmacy.com/en/calpol-sixplus-suspension-sugar-free-strawberry-flavour-6-years-80ml> |
| Bonjela ^a^ | Cost per tube | 2.99 | Parent/Questionnaire/<http://www.lloydspharmacy.com/en/bonjela-teething-gel-15g> |
| Anbesol ^a^ | Cost per tube | 2.39 | Parent/Questionnaire/<http://www.lloydspharmacy.com/en/anbesol-teething-gel-6007901-44> |
| Clove oil ^a^ | Cost per bottle | 1.00 | Parent/Questionnaire/<http://www.lloydspharmacy.com/en/care-clove-oil-10ml> |
| Child’s soluble dispirin ^a^ | Cost per tablet | 0.11 | Parent/Questionnaire/<http://www.lloydspharmacy.com/en/disprin-aspirin-32-soluble-tablets> |

^a^ Liquid and gel medications were assumed to have a life span of at least 6 months to prevent double counting
