# Supplementary material for: Cost-effectiveness of child caries management: a randomised controlled trial (FiCTION trial)
Source: BMC Oral Health. 2020 Feb 10;20:45. doi: 10.1186/s12903-020-1020-1 (PMC7011536; doi:10.1186/s12903-020-1020-1)
Supplement: Supplementary file 2 — Additional file 2. “Groupings for referrals” is a table summarising the different groupings used to categorise patient referrals. [file 12903_2020_1020_MOESM2_ESM.docx]

**Additional File 2**

**Table** Groupings for referrals

| **Referral grouping** | **Referral Description** |
| --- | --- |
| **A** | Consultation only – 1 X ½ hour appointment at Dental Hospital for assessment (+/- radiographs) |
| **B** | GA 20 – 3 consultations – 1 X ½ hour appt at Dental Hospital (Specialist or Consultant) and radiographs, 1 appt at Hospital for General Anesthesia (20 mins) as day case for extractions, pediatric bed for the morning (preparation and recovery), 1 appt back with Dental Hospital for review. |
| **C** | GA 45 – 3 consultations – 1 X ½ hour appt at Dental Hospital (Specialist or Consultant) and radiographs, 1 appt at Hospital for General Anesthesia (45 mins) as day case with restorative care, pediatric bed for the morning (preparation and recovery), 1 appt back with Dental Hospital for review |
| **D** | Inhalation sedation treatment – 1X ½ hour at Dental Hospital Specialist or Consultant) and radiographs, 1-3 appts under sedation at Dental Hospital (½ to ¾ of an hour with a mixture of extractions and restorative treatment) (Specialist or Consultant carrying out treatment) with follow up appointment for review |
| **E** | Behavior management - 1 X ½ hr at Dental Hospital (Specialist or Consultant) and radiographs 1-4 appts for ½ hour behavior management with extractions or restorative treatment |
| **F** | Referral to Orthodontist (Specialist or Consultant) in Dental Hospital or Specialist Practice / Pediatric Dentist (Specialist or Consultant) for consultation on possible orthodontic treatment - 1 X ½ hour appt with radiograph |
