# Supplementary material for: Cost-effectiveness of child caries management: a randomised controlled trial (FiCTION trial)
Source: BMC Oral Health. 2020 Feb 10;20:45. doi: 10.1186/s12903-020-1020-1 (PMC7011536; doi:10.1186/s12903-020-1020-1)
Supplement: Supplementary file 3 — Additional file 3 “Cost-effectiveness analysis for the comparison of PA vs B+P vs C+P arms based on fee-for-service costs in Scotland only (n=287)” is a the results of a sensitivity analysis which estimates costs based on charges to the NHS, based on the Scottish reimbursement rates (fee-for-service). [file 12903_2020_1020_MOESM3_ESM.docx]

**Additional File 3**

**Table** Cost-effectiveness analysis for the comparison of PA vs B+P vs C+P arms based on fee-for-service costs in Scotland only (n=287)

| **Investigation strategy** | **Cost [£]**  **[97.5% CI]** | **Incremental Cost [£]**  **[97.5% CI]^a^** | **Incidence**  **[97.5% CI]** | **Incremental incidence**  **[97.5% CI]^a^** | | **ICER [£]** | **Probability of each strategy being considered cost-effective at different threshold values for society’s willingness to pay to avoid an incidence of dental pain and/or infection** | | | | |
| --- | --- | --- | --- | --- | --- | --- | --- | --- | --- | --- | --- |
| **Incremental cost per incidence of dental pain and/or infection avoided** | | | | | | | **£0** | **£50** | **£100** | **£250** | **£500** |
| **PA (n=97)** | 321.46  [282 to 360] |  | 0.423  [0.31 to 0.54] |  | |  | 0.98 | 0.98 | 0.97 | 0.91 | 0.69 |
| **C+P (n=90)** | 359.28  [310 to 408] | 34.89  [-6 to 76] | 0.442  [0.33 to 0.56] | 0.016  [-0.14 to 0.17] | | **Dominated by PA** | 0.02 | 0.02 | 0.03 | 0.04 | 0.22 |
| **B+P (n=100)** | 374.54  [324 to 425] | 52.84  [12.79 to 93] | 0.37  [0.26 to 0.48] | -0.052  [-0.20 to 0.10] | | **1016.15^c^** | 0.00 | 0.00 | 0.00 | 0.05 | 0.09 |
|  | | | | | | | | | | | |
| **Investigation strategy** | **Cost [£]**  **[97.5% CI]** | **Incremental Cost [£]**  **[97.5% CI]^b^** | **Episode**  **[97.5% CI]** | | **Incremental episode**  **[97.5% CI]^b^** | **ICER [£]** | **Probability of each strategy being considered cost-effective at different threshold values for society’s willingness to pay to avoid an episode of dental pain and/or infection** | | | | |
| **Incremental cost per episode of dental pain and/or infection avoided** | | | | | | | **£0** | **£50** | **£100** | **£250** | **£500** |
| **PA (n=97)** | 321.46  [282 to 360] |  | 0.706  [0.47 to 0.94] |  | |  | 0.98 | 0.94 | 0.81 | 0.38 | 0.13 |
| **C+P (n=90)** | 359.28  [310 to 408] | 34.89  [-6 to 76] | 0.586  [0.40 to 0.77] | -0.126  [-0.39 to 0.14] | | **276.90** | 0.02 | 0.05 | 0.13 | 0.26 | 0.25 |
| **B+P (n=100)** | 374.54  [324 to 425] | 17.95  [-59 to 23] | 0.478  [0.31 to 0.64] | -0.097  [-0.36 to 0.17] | | **185.05** | 0.00 | 0.01 | 0.06 | 0.36 | 0.62 |
| **B+P vs PA** |  | 52.84  [12.79 to 93] |  | -0.223  [-0.49 to 0.04] | | **236.95** | 0.00 | 0.01 | 0.08 | 0.50 | 0.82 |

^a^ estimated based on adjusted analysis (n=287); ICER = incremental cost-effectiveness ratio; ^b^ ICER is estimated as B+P vs PA
