# Supplementary material for: Cost-effectiveness of child caries management: a randomised controlled trial (FiCTION trial)
Source: BMC Oral Health. 2020 Feb 10;20:45. doi: 10.1186/s12903-020-1020-1 (PMC7011536; doi:10.1186/s12903-020-1020-1)
Supplement: Supplementary file 4 — Additional file 4. “Cost-effectiveness analysis for the comparison of PA vs B+P vs C+P arms based on units of dental activity in England and Wales only costs (n=771)” is a the results of a sensitivity analysis which estimates costs based on charges to the NHS, based on the English reimbursement rates (Units of Dental Activity). [file 12903_2020_1020_MOESM4_ESM.docx]

**Additional File 4**

Table Cost-effectiveness analysis for the comparison of PA vs B+P vs C+P arms based on units of dental activity in England and Wales only costs (n=771)

| **Investigation strategy** | **Cost [£]**  **[97.5% CI]** | **Incremental Cost [£]**  **[97.5% CI]^a^** | **Incidence**  **[97.5% CI]** | **Incremental incidence**  **[97.5% CI]^a^** | **ICER [£]** | **Probability of each strategy being considered cost-effective at different threshold values for society’s willingness to pay to avoid an incidence of dental pain and/or infection** | | | | |
| --- | --- | --- | --- | --- | --- | --- | --- | --- | --- | --- |
| **Incremental cost per episode of dental pain and/or infection avoided** | | | | | | **£0** | **£50** | **£100** | **£250** | **£500** |
| **PA (n=257)** | 219.19  [201 to 237] |  | 0.452  [0.38 to 0.52] |  |  | 1.00 | 1.00 | 1.00 | 1.00 | 0.79 |
| **C+P (n=262)** | 286.71  [266 to 307 | 59.67  [35 to 84] | 0.398  [0.33 to 0.46] | -0.063  [-0.16 to 0.03] | **947.14** | 0.00 | 0.00 | 0.00 | 0.00 | 0.12 |
| **B+P (n=252)** | 291.56  [269 to 314] |  | 0.398  [0.33 to 0.47] |  | **Dominated by C+P** | 0.00 | 0.00 | 0.00 | 0.00 | 0.09 |
|  | | | | | | | | | | |
| **Investigation strategy** | **Cost [£]**  **[97.5% CI]** | **Incremental Cost [£]**  **[97.5% CI]^a^** | **Episode**  **[97.5% CI]** | **Incremental episode**  **[97.5% CI]^a^** | **ICER [£]** | **Probability of each strategy being considered cost-effective at different threshold values for society’s willingness to pay to avoid an episode of dental pain and/or infection** | | | | |
| **Incremental cost per episode of dental pain and/or infection avoided** | | | | | | **£0** | **£50** | **£100** | **£250** | **£500** |
| **PA (n=257)** | 219.19  [201 to 237] |  | 0.700  [0.47 to 0.75] |  |  | 1.00 | 1.00 | 1.00 | 0.83 | 0.39 |
| **C+P (n=262)** | 286.71  [266 to 307 | 59.67  [35 to 84] | 0.610  [0.47 to 0.75] | -0.106  [-0.29 to 0.08] | **562.92** | 0.00 | 0.00 | 0.00 | 0.09 | 0.33 |
| **B+P (n=252)** | 291.56  [269 to 314] | 5.85  [-19 to 31] | 0.599  [0.47 to 0.73] | -.006  [-0.19 to 0.18] | **975** | 0.00 | 0.00 | 0.00 | 0.08 | 0.28 |

^a^ estimated based on adjusted analysis (n=770); ICER = incremental cost-effectiveness ratio
