# Supplementary material for: Cost-effectiveness of child caries management: a randomised controlled trial (FiCTION trial)
Source: BMC Oral Health. 2020 Feb 10;20:45. doi: 10.1186/s12903-020-1020-1 (PMC7011536; doi:10.1186/s12903-020-1020-1)
Supplement: Supplementary file 5 — Additional file 5. “Cost-effectiveness analysis for the comparison of PA vs B+P vs C+P based on units of dental activity (England and Wales) and fee-for service (Scotland) costs (n=1058)” is a the results of a sensitivity analysis which estimates costs based on charges to the NHS, based on the both the Scottish and English reimbursement rates. [file 12903_2020_1020_MOESM5_ESM.docx]

**Additional File 5**

Table Cost-effectiveness analysis for the comparison of PA vs B+P vs C+P based on units of dental activity (England and Wales) and fee-for service (Scotland) costs (n=1058)

| **Investigation strategy** | | **Cost [£]**  **[97.5% CI]** | **Incremental Cost [£]**  **[97.5% CI]^b^** | **Incidence**  **[97.5% CI]** | **Incremental incidence**  **[97.5% CI]^b^** | **ICER [£]** | **Probability of each strategy being considered cost-effective at different threshold values for society’s willingness to pay to avoid an incidence of dental pain and/or infection** | | | | |
| --- | --- | --- | --- | --- | --- | --- | --- | --- | --- | --- | --- |
| **Incremental cost per incidence of dental pain and/or infection avoided** | | | | | | | **£0** | **£50** | **£100** | **£250** | **£500** |
| **PA** | | 247.21  [230 to 265] |  | 0.410  [0.35 to 0.47] |  |  | 1.00 | 1.00 | 1.00 | 1.00 | 0.88 |
| **C+P** | | 305.26  [285 to 325] | 50.91  [28 to 73] | 0.410  [0.35 to 0.47] | -0.043  [-0.12 to 0.04] | **1183.95** | 0.00 | 0.00 | 0.00 | 0.00 | 0.06 |
| **B+P** | | 315.26  [293 to 337] | 10.71  [-11 to33] | 0.390  [0.33 to 0.45] | -0.016  [-0.10 to 0.06 | **669.38** | 0.00 | 0.00 | 0.00 | 0.00 | 0.06 |
| **B+P vs PA** | |  | 61.62  [39 to 84] |  | -0.058  [-0.14 to 0.02] | **1062.41** | 0.00 | 0.00 | 0.00 | 0.00 | 0.07 |
|  | | | | | | | | | | | |
| **Investigation strategy** | | **Cost [£]**  **[97.5% CI]** | **Incremental Cost [£]**  **[97.5% CI]^b^** | **Episode**  **[97.5% CI]** | **Incremental episode**  **[97.5% CI]^b^** | **ICER [£]** | **Probability of each strategy being considered cost-effective at different threshold values for society’s willingness to pay to avoid an episode of dental pain and/or infection** | | | | |
| **Incremental cost per episode of dental pain and/or infection avoided** | | | | | | | **£0** | **£50** | **£100** | **£250** | **£500** |
| **PA** | 247.21  [230 to 265] | |  | 0.701  [0.58 to 0.82] |  |  | 1.00 | 1.00 | 1.00 | 0.80 | 0.29 |
| **C+P** | 305.26  [285 to 325] | | 50.91  [28 to 73] | 0.603  [0.49 to 0.71] | -0.111  [-0.26 to 0.04] | **458.65** | 0.00 | 0.00 | 0.00 | 0.11 | 0.32 |
| **B+P** | 315.26  [293 to 337] | | 10.71  [-11 to33] | 0.565  [0.46 to 0.67] | -0.031  [-0.18 to 0.12] | **357** | 0.00 | 0.00 | 0.00 | 0.09 | 0.39 |
| **B+P vs PA** |  | | 61.62  [39 to 84] |  | -0.143  [-0.26 to 0.01] | **430.91** | 0.00 | 0.00 | 0.00 | 0.10 | 0.57 |

^a^ estimated based on adjusted analysis (n=1057); ICER = incremental cost-effectiveness ratio
