# Supplementary figures and images for: Cost-effectiveness of child caries management: a randomised controlled trial (FiCTION trial)
Source: BMC Oral Health. 2020 Feb 10;20:45. doi: 10.1186/s12903-020-1020-1 (PMC7011536; doi:10.1186/s12903-020-1020-1)

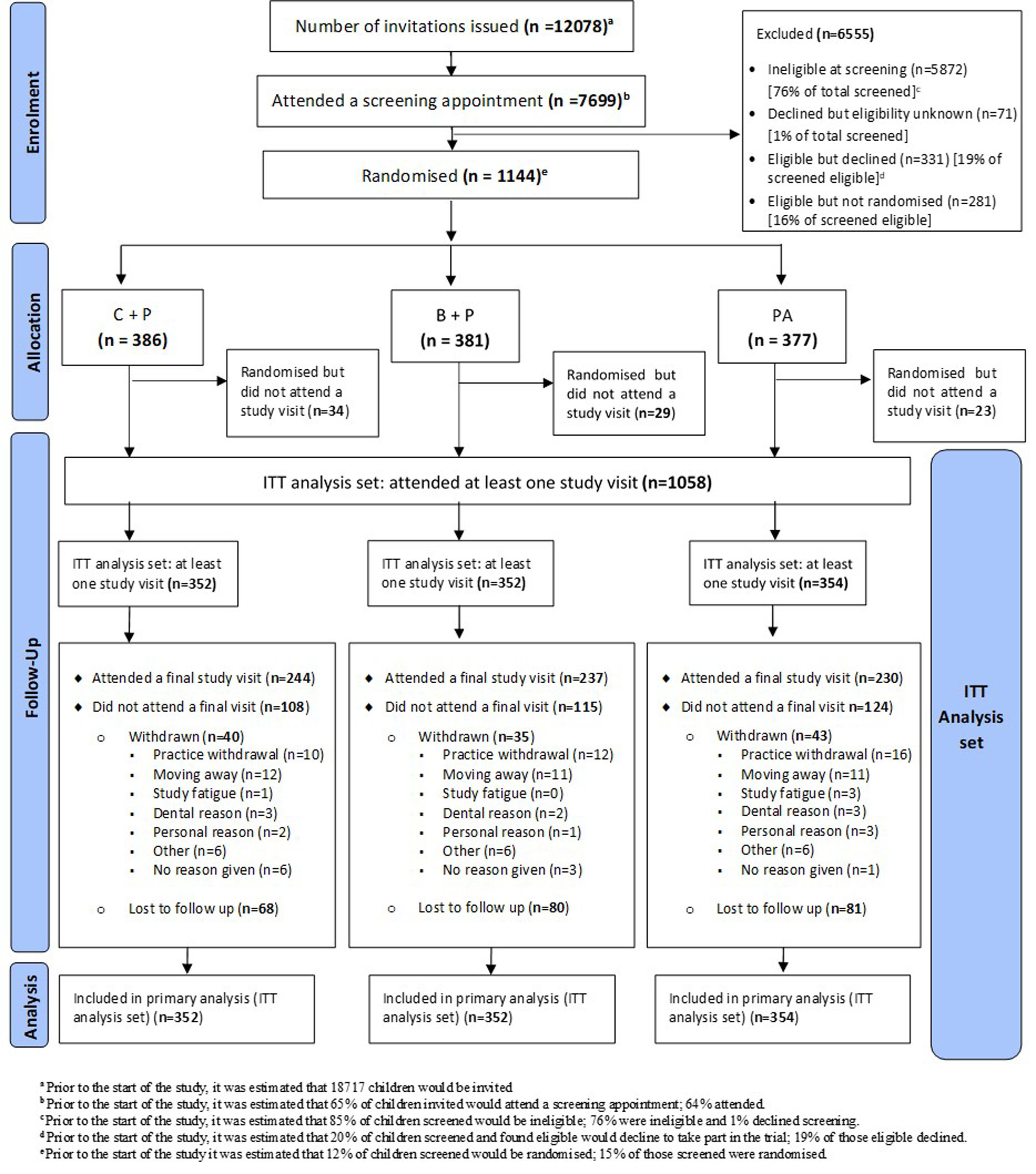

Supplement: Supplementary file 6 — Additional file 6. “CONSORT flow diagram of participant journey through trial” illustrates the number of children screened, randomised, and included in the final analysis. This image is taken directly from Maguire et al. 2019. [file 12903_2020_1020_MOESM6_ESM.jpg]
